# Respiration recording for fMRI: breathing belt versus spine coil sensor

**DOI:** 10.1162/imag_a_00239

**Published:** 2024-07-24

**Authors:** Marilena Wilding, Anja Ischebeck, Natalia Zaretskaya

**Affiliations:** Department of Psychology, University of Graz, Graz, Austria; BioTechMed-Graz, Graz, Austria

**Keywords:** fMRI, physiological noise modelling, RETROICOR, breathing belt, spine coil sensor, respiration

## Abstract

Physiological signals such as pulse and respiration strongly contribute to non-neuronal signal change of the blood oxygenation level-dependent (BOLD) contrast in functional magnetic resonance imaging (fMRI). This has been observed not only during task-based but also during resting-state fMRI measurements, where the confounding influence of physiological signals is most pronounced. Over the last decades, a variety of techniques evolved, aiming at detecting and removing physiological artifacts in fMRI time series. These follow either a solely data-driven approach or rely on externally recorded physiological data. To record cardiac and respiratory signals, typically pulse oximetry or electrocardiography (ECG) and a respiration belt are used, respectively. New technologies allow to capture respiratory signal directly with a sensor placed within the spine coil in the patient table, eliminating the need of a respiration belt, which considerably increases participants’ comfort. However, little is known about the effectiveness of these new technologies and how they compare to the standard respiration belt recording. In the current study, we compared the two devices, respiration belt and spine coil sensor, in their suitability for physiological noise removal during a visual perception task and during rest. We did not find any differences in resting-state functional connectivity (RSFC), stimulus-related activity, or residual noise between data corrected with the two recording devices. Our results show that spine coil-derived respiration recordings are as good as belt respiration recordings for physiological noise removal in task-induced activity, with spine coil recordings having an additional advantage in terms of participants’ comfort and artifact susceptibility.

## 1 Introduction

The blood oxygenation level-dependent (BOLD) contrast is one of the most frequently used indicators of brain activity in human neuroimaging. Although it is an indirect measure of neuronal events, it, nevertheless, reflects changes in the oxygenation of the blood that are evoked by neuronal activity. This signal, however, does not only indicate neuronal activity. It further contains several confounding noise components that obscure the main signal. Multiple noise sources have been identified over the last decades, such as background noise, thermal noise (system-related noise), instrumental drifts, instabilities of hardware, participant’s head motion as well as physiological signals, such as cardiac rate, respiratory rate, and resultant changes in the level of CO_2_(Caballero-Gaudes & Reynolds, 2017;Keilholz et al., 2017;Liu, 2016).

Participant motion and physiological noise have been found to contribute most to non-neuronal signal change in the BOLD contrast (Caballero-Gaudes & Reynolds, 2017;Kasper et al., 2017;Keilholz et al., 2017). Studies suggest its contribution to the signal variance to be comparable to that of the BOLD signal fluctuations during a task and at rest (Caballero-Gaudes & Reynolds, 2017). Task-based fMRI is thought to be more robust to non-neuronal noise since the observed effects are usually a result of averaging over multiple trials. However, this view fails to consider the notion that changes in cardiac rate or breathing rate can correlate with the examined task, especially when it is emotionally arousing or cognitively challenging, and hence introduce additional noise in a task-locked fashion (Birn et al., 2009;Bright et al., 2014). In resting-state functional connectivity (RSFC) analysis, the influence of physiological noise is even more pronounced, as non-neuronal noise can introduce spurious correlations between voxels’ time series (Birn, 2012;Murphy et al., 2013), which can even mimic functional brain networks (Bright et al., 2020;Chen et al., 2020). Although physiological noise is typically observed at frequencies higher than the usual BOLD signal fluctuations at rest (i.e., cardiac: ~1 Hz, respiratory: ~0.3 Hz, BOLD: <0.1 Hz), due to aliasing effects present at repetition times (TRs) of ≥2 s, the physiological signals can still appear in the low-frequency segment of the power spectrum, where they are hard to distinguish from low-frequency oscillations of neural origin (Bhattacharyya & Lowe, 2004;Murphy et al., 2013).

### 1.1 Effects of cardiac and respiratory noise

Cardiac pulsation activity is thought to have a substantial effect on the relative distribution of fluid components (e.g., cerebrospinal fluid, blood) and also more solid brain tissue within the skull (Bhattacharyya & Lowe, 2004;Dagli et al., 1999). Expectedly, its effects are most noticeable near major blood vessels, as an increase in blood pressure intensifies pulsation of the vessels and hence causes local movements (Dagli et al., 1999;Glover et al., 2000). Changes in the cardiac rate were also found to be related to variations in the BOLD signal amplitude, which is not surprising since both signals are dependent on cerebral blood flow (CBF), cerebral blood volume (CBV), and oxygenation. This close relationship, however, can introduce confounds not only around large vessels, but also within the gray matter tissue (Chang et al., 2009;Shmueli et al., 2007).

Respiratory activity can affect the BOLD signal in various ways. First, abdominal motion can lead to perturbation of the magnetic field (by inducing changes in field strength and homogeneity), which can cause distortion of the acquired data (Raj et al., 2001). Small head movements during breathing can further produce spin history effects that can last for several volumes and are spatially dependent on the axis of the movement (Caballero-Gaudes & Reynolds, 2017;Friston et al., 1996;Muresan et al., 2005). In addition to effects related to motion, even small and ordinary variations in respiratory volume and rate can induce considerable changes in the low-frequency BOLD signal. This is due to the fact that changes in breathing are accompanied by shifts in the arterial level of carbon dioxide (CO_2_), which, as an important vasodilator, modulates local cerebral blood flow (Birn et al., 2006;Wise et al., 2004). This is especially relevant near locations with high blood volume, such as blood vessels and gray matter (Birn et al., 2008). Together, these findings highlight the complexity of distinguishing signal driven by neural activity from mere physiological noise, which can affect the BOLD signal in a broad variety of ways.

### 1.2 Minimising physiological noise

Multiple techniques to determine and, most importantly, remove physiologically induced noise in BOLD time series have evolved over the last years (Agrawal et al., 2020;Caballero-Gaudes & Reynolds, 2017;Murphy et al., 2013), choosing one of the following approaches or their combination. On the one hand, data-driven models aim to approximate the contribution of physiological fluctuations based on signals from regions which are not likely to be effectively influenced by neuronal activity, such as cerebrospinal fluid (CSF) and white matter. These estimations, however, only allow to remove the average signal of said regions, approximating physiological contribution to gray matter signal and fitting it to every gray matter voxel. Based on this principle, more advanced methods such as CompCor (component-based noise correction method) have been developed, thus enabling modelling the physiological signal in gray matter as multiple principle components of the CSF and white matter time courses instead of using the average signal (Behzadi et al., 2007).

Reference-based approaches, on the other hand, aim at estimating the physiological noise not from indirect sources but from data acquired using external measurement devices (such as pulse oximeter, respiration belt, or electrocardiogram), which directly record physiological signals throughout the fMRI session. These signals are then retrospectively synchronised with the functional data (Kasper et al., 2017) in order to estimate the influence of physiology on each voxel’s time series. One widely established example is the RETROICOR (retrospective image correction) algorithm, which aims to determine the phase of each cardiac and respiratory cycle whenever a slice is acquired. Subsequently, the periodic effects of the physiological noise are modelled using a Fourier expansion of the phases that is fit to the time series of each voxel (Glover et al., 2000).

Despite the substantial effects of respiration on the BOLD signal (Birn et al., 2008;Caballero-Gaudes & Reynolds, 2017;Chang et al., 2009;Liu, 2016;Murphy et al., 2013;Verstynen & Deshpande, 2011), little is known about the different means of physiological signal recording and their suitability for subsequent physiological signal modelling. Traditionally, respiratory activity is recorded using a respiration belt that is applied around an individual’s upper torso near the diaphragm and registers the shift in abdominal volume. The new BioMatrix technology implemented in more recent models of the Siemens MRI scanners, however, additionally allows the recording of respiration via a sensor embedded in the spine coil within the patient table (Runge et al., 2019). The sensor produces a local magnetic field that changes with respiration-induced motion. This option is fully integrated into the scanner’s structure and hence eliminates the necessity for respiration belt usage, which increases participants’ comfort. Since the spine coil sensor does not require any setup, this may be another advantage in terms of artifact susceptibility. In contrast, during belt recording, movements of the torso or high respiratory volume can disrupt the normal recording and introduce corrupted signal. Whether such differences would be significant enough to be reflected in the overall data quality, however, is not clear. Therefore, it is generally unclear whether the new spine coil technology reflects the respiratory signal in a comparable manner and is equally suitable for modelling physiological artifacts in fMRI experiments as the breathing belt.

To investigate this, we simultaneously recorded the two types of signals in one task-based and one resting-state fMRI dataset, and formally compared them in their suitability for reducing physiological noise.

## 2 Materials and Methods

### 2.1 Participants

The task-based dataset comprised 25 participants between 19 and 31 years (12 female, mean age = 23.61, SD = 3.40) and the resting-state dataset included 56 volunteers between 19 and 35 years (35 female, mean age = 24.22, SD = 3.15) after excluding 6 and 4 participants, respectively, either due to insufficient physiological signal quality (3 task-based and 3 resting-state) or technical issues during physiological signal recording (3 task-based and 1 resting-state). Participants had no neurological, psychiatric, or cardiovascular diseases and were not taking any medication on a regular basis. Before data acquisition, they gave written informed consent and were instructed in a standardised manner. Both studies were conducted following the Declaration of Helsinki and were approved by the local ethics committee of the University of Graz. Detailed descriptions of the two datasets are provided in our previous studies (task:Wilding et al., 2022, rest:Wilding et al., 2023).

### 2.2 Data acquisition

#### 2.2.1 fMRI data

fMRI data for both datasets were acquired on a 3T Siemens Magnetom Vida scanner (Siemens Healthineers, Erlangen, Germany) using a 64-channel head coil. Functional images were obtained using the blood oxygenation level-dependent (BOLD) contrast. For detailed acquisition parameters, seeTable 1.

**Table 1.** fMRI acquisition parameters for both datasets.

	Rest	Task
TR	3.22 s	0.88 s
TE	32 ms	30 ms
Multiband factor	-	3
Acceleration factor	-	GRAPPA, 2
Voxel size	3 x 3 x 3 mm	3 x 3 x 3 mm
Flip angle	82°	65°
FOV	228 mm	210 mm
Number of slices	46	45
Number of volumes	155	On avg. 5100

##### 2.2.1.1 Rest

The resting-state dataset was acquired in an 8-minute scanning session. Participants were instructed to fixate a red dot in the centre of the screen and avoid any directed cognition. Resting-state measurements were followed by several task runs, which are not discussed in this paper.

##### 2.2.1.2 Task

Task-related data were acquired using a multiband echo planar imaging sequence (EPI). During the experiment, participants were repeatedly presented with a bistable motion stimulus for a duration of 1 s, interleaved with long (25-50 s) baseline periods. Immediately after the stimulus presentation they had to report their spontaneously occurring percept via a button press. Each participant underwent 6 functional runs of around 13 minutes, with the exact duration depending on the randomisation of the inter-stimulus periods. This resulted in a total measuring time of approximately 75 minutes.

#### 2.2.2 Physiological data

Physiological measures in both studies were recorded throughout the whole fMRI data acquisition period. Heart rate was recorded with a photoplethysmograph clip at the participants’ left index finger at a sampling rate of 400 Hz. This technique allows to monitor and record an individual’s pulse by measuring the absorption of infrared light in body tissue, which is modulated by the cardiac cycle (Nilsson, 2013).

The respiration signal was captured simultaneously by two devices, the respiration belt (*belt*) and the spine coil sensor (*spine*). The respiration belt was placed around the upper torso. An air cushion was placed between the belt and the torso that detected pressure changes induced by the participant’s chest contraction and expansion (sampling rate: 400 Hz). The spine coil respiration sensor (also 400 Hz sampling rate) was embedded in the BioMatrix spine coil of the Siemens Magnetom Vida scanner (Runge et al., 2019). It applied a local electromagnetic field near the torso, which differed from the Larmor frequency (i.e., 30 MHz) and hence did not interfere with the main scanner signal. This electromagnetic field varied with motion produced by the different phases of the respiratory cycle. Surrounding coil elements detected these changes, which reflected the participant’s breathing pattern. The recording of physiological signals was performed using the command line tool*ideacmdtool*by logging pulse and respiration signals throughout the fMRI data acquisition. This resulted in two files, one containing pulse and one containing respiration data, which were used for further processing.

### 2.3 Data analysis

#### 2.3.1 Raw signal correspondence

First, we formally examined the correspondence between the raw respiratory signals acquired with*belt*and*spine*for both datasets. We calculated cross-correlation between the two signals over the whole run, examining the maximal correlation over lags. By computing cross-correlation, we were also able to identify the lag between the two signals (seeFig. 1Aand1B) of every subject by examining the lag at which the cross-correlation value is maximal.

**Fig. 1.**
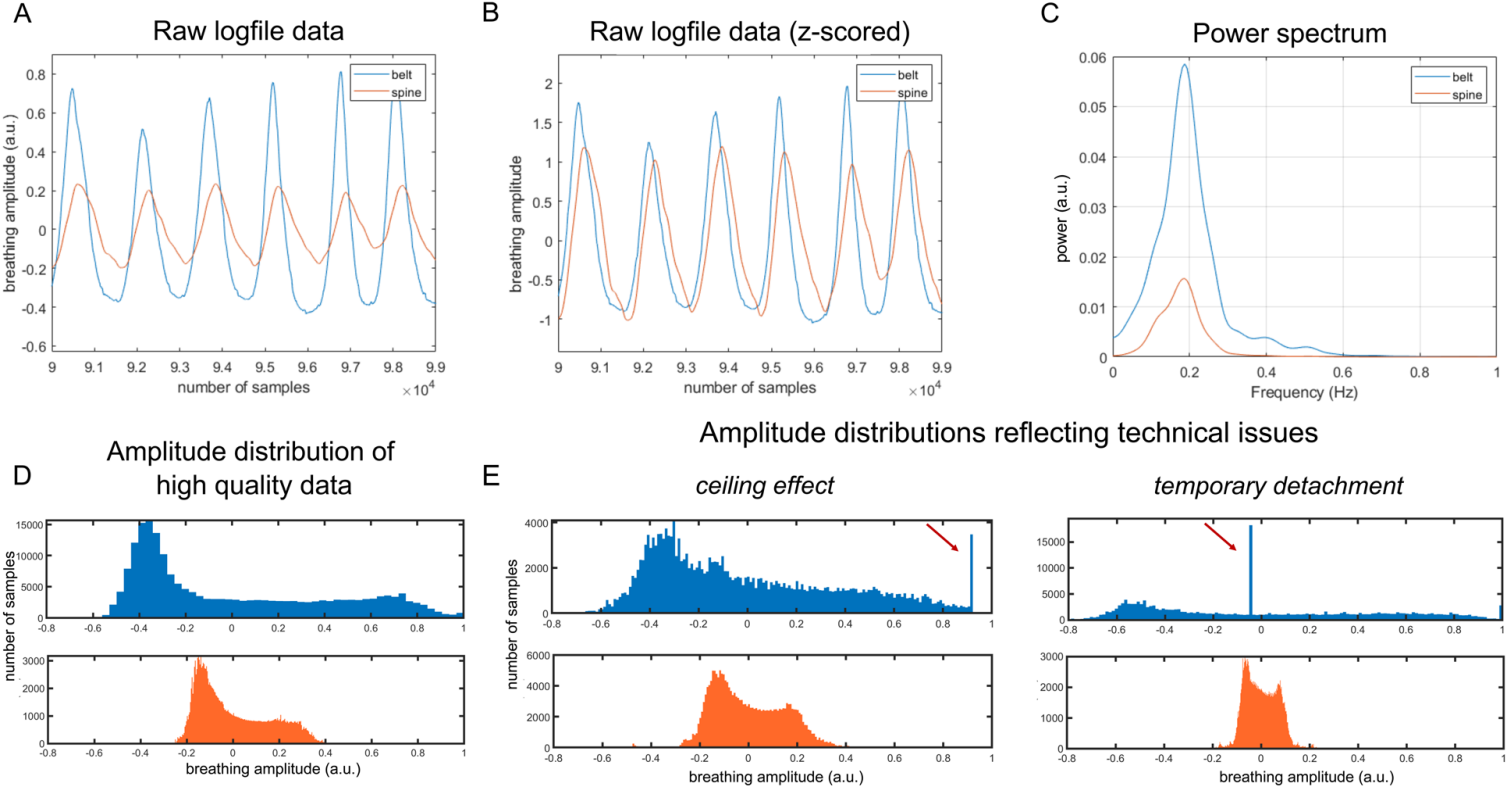
Comparison of respiratory signal captured with a breathing belt (*belt*; blue) or spine coil sensor (*spine*; orange) for an exemplary subject. (A) Example raw signal for both recordings showing a few breathing cycles. (B) Z-transformed amplitudes of both devices shown in A for a few breathing cycles. (C) Power spectrum of the two signals. (D) Typical distribution of amplitudes in the raw signal for both recordings, both following a right-skewed distribution. (E) Example of typical problems during the respiratory signal recording (indicated by red arrows) reflecting ceiling effects (belt was too tight; for details see SupplementaryFig. S5) and temporary detachment of the belt, which are visible only in the*belt*recording. More individual raw data examples and power spectra are shown in SupplementaryFigures S1, S2, and S3.

#### 2.3.2 Processing of the physiological data

Physiology regressors were obtained from the physiological log-files using the MATLAB-based*PhysIO*toolbox (Kasper et al., 2017). In the first step, respiratory data quality was checked by inspecting raw data (Fig. 1A) as well as the distribution of breathing amplitudes, allowing us to examine signal quality and detect potential issues with signal recording. Sufficient respiratory signal quality was reflected in a high number of low/intermediate amplitudes with a long tail toward higher amplitudes, which correspond to sporadic deep breaths (Fig. 1D). In the case of technical problems during recording, such as ceiling effects in the respiratory signal (e.g., belt was too tight) or prolonged signal loss (e.g., temporary detachment of belt, seeFig. 1E), there is a distribution peak at the maximum amplitude or around zero, respectively. Cardiac signal was examined for possible periods of detachment by visually inspecting the data. Next, the cardiac and respiratory signal was aligned with the fMRI time series using the system time stamps and an algorithm performing iterative peak detection was applied to identify repetitive signal features and discard compromised segments and noise. Problematic time series were excluded when artifactual signal segments were present in ≥40 % of the signal, which was the case in three participants of each dataset. The remaining data were processed with RETROICOR phase expansion algorithm (Glover et al., 2000), which allows to consider variation within the physiological signals by modelling the periodic effects of pulsatile motion and field fluctuations as Fourier expansion of the respiratory phase. The expansion orders were set following the parameters ofHarvey et al. (2008), that is, 3rd-order cardiac model, 4th-order respiratory model, and 1st-order interaction model. This resulted in 6 cardiac phase regressors, 8 respiratory phase regressors, and 4 interaction terms; in total 18 regressors per device (RETROICOR*_belt_*and RETROICOR*_spine_*). Finally, regressors were downsampled to a respective acquisition TR (see SupplementaryFig. S4for examples) and added to the General Linear Model (GLM).

Signal correspondence of the unprocessed respiratory signal recorded using*belt*and*spine*was quantified using cross-correlation (*xcorr*function) in MATLAB R2019b (MathWorks, Inc., Natick, MA).

#### 2.3.3 Preprocessing of fMRI data

Structural and functional data of both datasets were preprocessed using fMRIPrep version 20.2.3 (Esteban et al., 2019), which is based on Nipype 1.6.1 (Gorgolewski et al., 2011). The description of preprocessing steps is based on the fMRIPrep boilerplate text.

##### 2.3.3.1 Anatomical data

The T1-weighted image was corrected for intensity non-uniformity with ANTs*N4BiasFieldCorrection*function and used as T1w-reference throughout the following steps. Next, the T1w-reference was skull-stripped and brain tissue segmentation into cerebrospinal fluid (CSF), white matter (WM), and gray matter (GM) was performed on the brain-extracted T1-weighted image using FSL*fast*function.

##### 2.3.3.2 Functional data

First, a reference volume and its skull-stripped version were generated. Next, a B0-nonuniformity map was estimated based on echo-planar imaging (EPI) references with opposing phase-encoding directions. Based on the estimated susceptibility distortion, a corrected EPI reference was calculated for a more accurate co-registration with the anatomical reference. The BOLD reference was co-registered to the T1w reference by applying boundary-based registration (*bbregister;*Greve & Fischl, 2009) with 6 degrees of freedom. Head-motion parameters with respect to the BOLD reference (transformation matrices for the six rotation and translation parameters) were estimated before any spatiotemporal filtering using*mcflirt*(FSL 5.0.9). After applying slice-timing correction, the BOLD time series were resampled onto MNI space by applying a single, composite transform to correct for head motion and susceptibility distortions.

The preprocessed datasets were then imported into the CONN toolbox (Whitfield-Gabrieli & Nieto-Castanon, 2012; version CONN21.a), which uses SPM 12 (http://fil.ion.ucl.ac.uk/spm/), and analysed separately.

#### 2.3.4 Resting-state data analysis

Resting-state data analysis was performed using CONN (version CONN21.a), which uses SPM 12. Functional data were smoothed using spatial convolution with a Gaussian kernel of 6 mm full width half at maximum (FWHM). The first four volumes of each run were discarded to allow for T1 equilibration effects. Potential outlier scans were identified using artifact detection software (ART) as volumes with framewise displacement above 0.9 mm or global BOLD signal changes above 5 SD. Next, the functional time series were denoised using a standard denoising pipeline (Nieto-Castanon, 2020), including the regression of potential confounding effects characterised by motion parameters derived from fMRIPrep (6) and their 1st-order derivatives (6), outlier scans (15 regressors or less, depending on the participant), and physiology regressors derived from the RETROICOR model (18). This was followed by bandpass filtering of the BOLD time series between 0.008 and 0.09 Hz. To compare the effect of the respiration recording method on the resting-state data, functional runs of each subject were added three times as three separate “sessions” that only differed in the type of physiology regressors (RETROICOR*_belt_,*RETROICOR*_spine_*or*none*).

##### 2.3.4.1 Functional connectivity analysis

To characterise the patterns of functional connectivity, ROI-to-ROI connectivity matrices (RRC) were estimated using the 164 HCP-ICA network parcellation, which is implemented in CONN. These matrices reflect the strength of functional connectivity between each pair of ROIs. Functional connectivity strength was represented by bivariate Fischer-transformed Pearson’s correlation coefficients, defined separately for each pair of target areas. First, we compared resting-state functional connectivity from data that was not corrected for physiological noise to data corrected with either*belt*or*spine*. In the next step, we directly compared functional connections in data corrected with*belt*or*spine*. Results were thresholded using a connection-level threshold of p < 0.01, and a cluster-level false discovery rate (FDR)–corrected threshold of p < 0.05. FDR correction was used because it is the most appropriate choice for ROI-to-ROI connectivity results since the random field theory cannot be applied to connection-level data.

##### 2.3.4.2 Residual noise analysis in the resting-state data

To examine differences in remaining noise after physiology correction with either recording, we compared the standard deviation of residuals between both. The denoised signal files were automatically created by the CONN toolbox after denoising, and the standard deviation of residual voxel time course over time was calculated for each 4-D volume using the*fslmaths*command in FSL version 6.0.4 (Smith et al., 2004). To evaluate the general effects of physiological noise modelling compared to no physiology correction, we performed paired t-tests between*none*and*belt/spine*using the respective standard deviation maps in SPM 12. To directly compare residual noise in the*belt*- and*spine*-corrected data, a voxel-wise paired t-test was calculated between*belt*and*spine*datasets. We also repeated all comparisons using adjusted R^2^of the model fits instead of the standard deviation of the residuals. This allowed us to account for differences in the number of regressors in the none versus belt/spine modes. The results were corrected for multiple comparisons using a threshold of p < 0.05, correcting for family-wise error rate (FWE).

#### 2.3.5 Task data analysis

Task-based data analysis was performed using the CONN toolbox (version 21.a), which uses SPM 12. The task-based functional data were smoothed using spatial convolution with a Gaussian kernel of 8 mm full width at half maximum. The first four volumes of each run were discarded to allow for T1 equilibration effects.

##### 2.3.5.1 Activity analysis

For the first-level analysis, stimulus onsets were modelled as events of 2 s duration as regressors of interest. In addition, motion parameters (6) and physiology regressors (18) were included into the GLM as nuisance regressors. The first-level GLM analysis was conducted twice, once with RETROICOR_belt_and once with RETROICOR_spine_nuisance regressors. Beta values for the stimulus onset of each GLM and each participant were used for the subsequent second-level group analysis. First, we examined stimulus-related activity in both models separately. To test whether there is an overall activity difference between*belt*and*spine*, a whole-brain analysis using the t-contrast “*belt*-*spine”*was performed on the second level. Results were thresholded using a voxel-level threshold of p < 0.01 and a cluster-level FWE-corrected threshold of 0.05.

##### 2.3.5.2 Residual noise analysis in the task data

To confirm our findings for the resting-state fMRI, we performed a similar analysis of residuals for the task-based fMRI dataset. To account for fluctuations related to task, in addition to including the noise regressors we also included the effects of task in the GLM before examining the standard deviation of the residuals. To directly compare*belt*and*spine*, we performed a voxel-wise paired t-test on the standard deviation maps using a threshold of p < 0.05, correcting for family-wise error rate (FWE).

All figures depicting functional results were created using the MATLAB-based*bspmview (v.20180918)*program (Spunt, 2016).

## 3 Results

### 3.1 Correspondence of signals

Over subjects, we observed a very high correspondence between the two signals (rest: r_max_= 0.89, SD = 0.09, IQR = 0.06; task: r_max_= 0.88, SD = 0.08, IQR = 0.06); seeFigure 2. The mean lag between two signals over all participants was 353 ms (SD = 0.09 ms) for the rest dataset and 350 ms (SD = 0.11 ms) for the task dataset (first run), with belt signal lagging behind the spine signal.

**Fig. 2.**
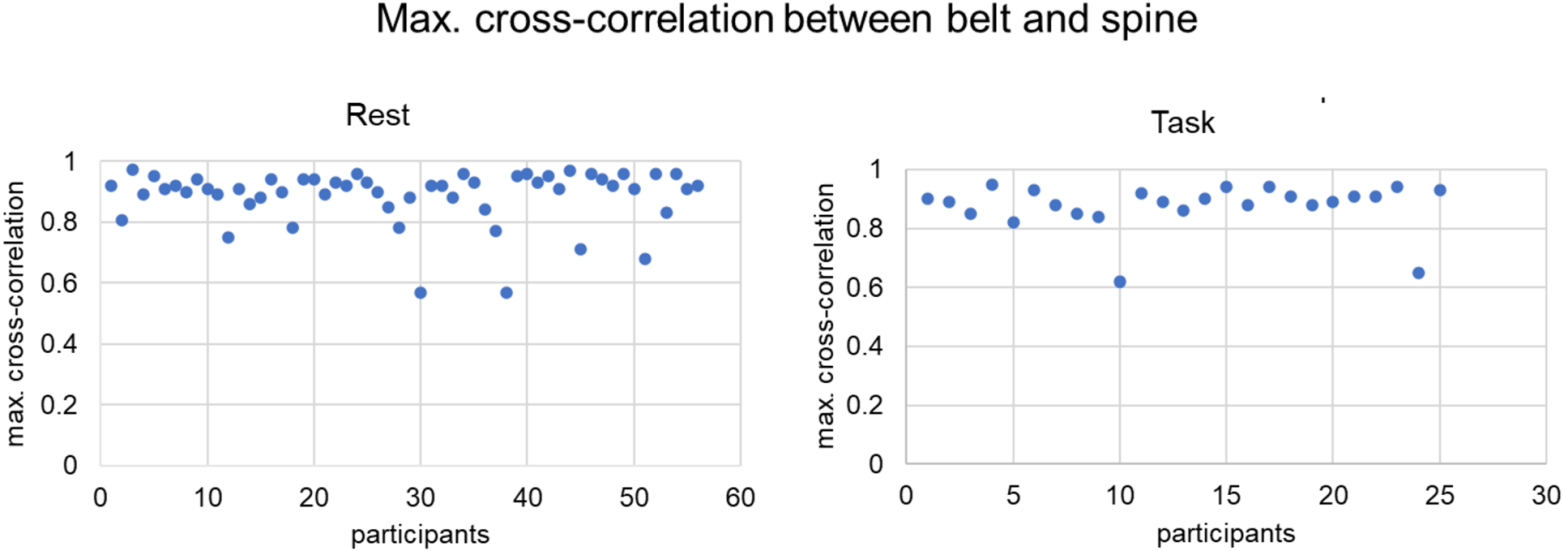
Maximal cross-correlation between respiratory signals recorded with belt and spine device for each participant, reported for both datasets. We observed high mean max. cross-correlations for both datasets (rest, r = 0.89, task (run 1), r = 0.88). SupplementaryFigure S4additionally demonstrates correspondence between signal-derived physiology regressors.

To further check whether the two signals exhibit any discrepancy in the frequency domain, we computed signal power spectra for several representative participants. We consistently observed both signals peaking around the typical respiratory frequency (0.2–0.3 Hz), and a higher power in belt compared to spine over a wide range of frequencies between 0 and 1 Hz. Power spectra of an example participant are shown inFigure 1Cand more examples in SupplementaryFigure S3.

The high correspondence between the signals is also reflected in the devices’ ability to record faster breathing patterns. In contrast to deep breaths, which may lead to signal clipping in the belt-derived recording (seeFig. 1Eand SupplementaryFig. S5), both methods performed well for recording faster breathing patterns. Based on the peaks of the power spectra, we selected three subjects with particularly fast breathing and examined the raw recording traces (SupplementaryFig. S6). No obvious issues were observed in these recordings.

### 3.2 Effects of noise correction

#### 3.2.1 Functional connectivity

**Fig. 3.**
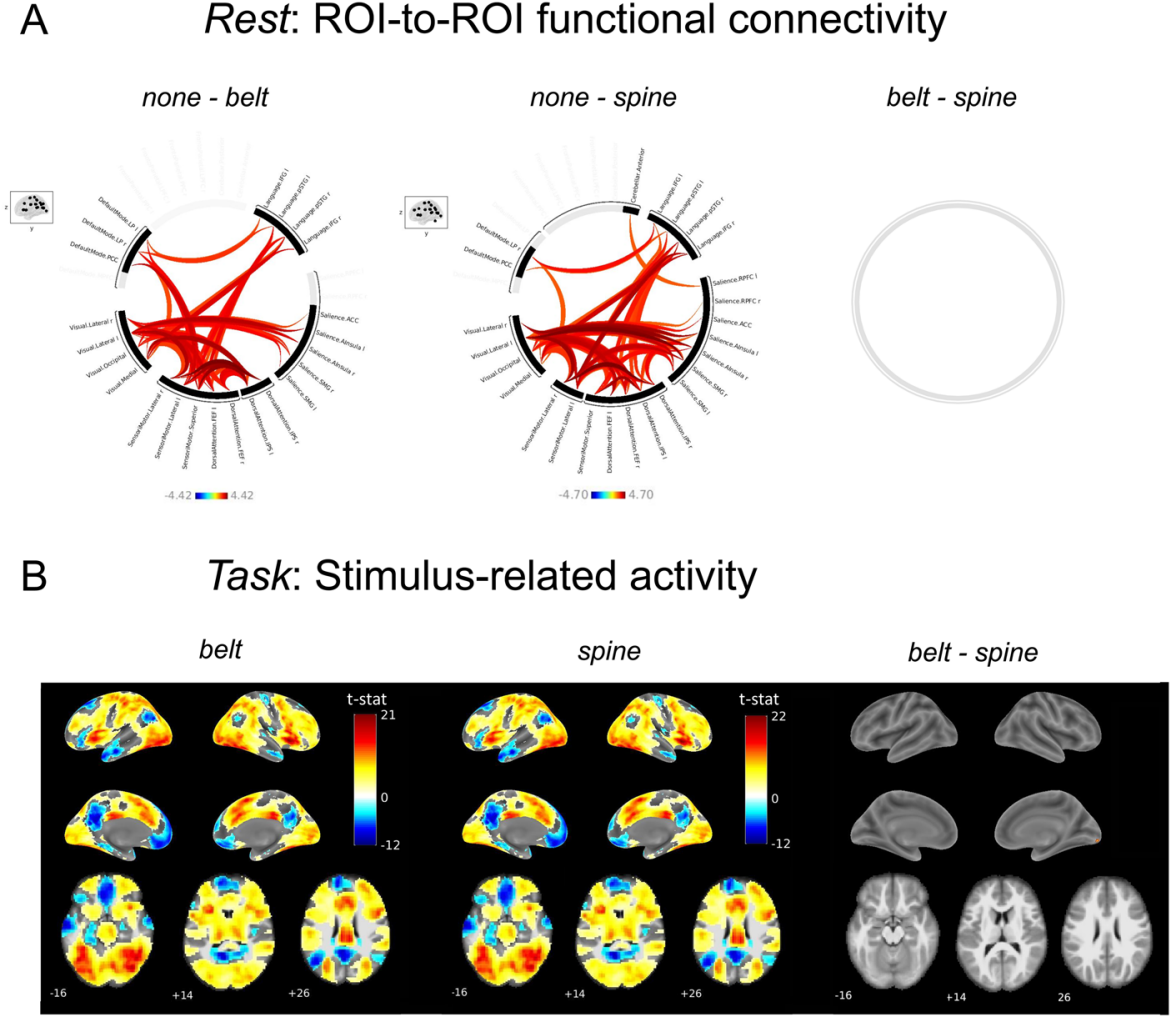
Effects of noise correction for both datasets. (A) ROI-to-ROI functional connectivity differences between*none*and*belt*(leftmost),*none*and*spine*(middle), and*belt*and*spine*(rightmost). Connection threshold: p < 0.01, cluster threshold: p < 0.05, FDR-corrected. (B) Stimulus-evoked activity for both types of denoising; voxel-threshold: p < 0.01, cluster-threshold: p < 0.05, FDR-corrected.

We examined the general effect of physiological noise modelling on functional connectivity by comparing functional connectivity derived from data denoised without physiology regressors (*none*) with functional connectivity corrected with either RETROICOR*_belt_*or RETROICOR*_spine_*. The comparison “*none*–*belt”*revealed significantly higher cortical ROI-to-ROI functional connectivity in the absence of physiological modelling, suggesting the presence of artifactual correlations produced by physiological noise (Fig. 3A). Similar results were found for the comparison “*none*–*spine.”*For the latter, we found a slightly higher number of significant functional connections than for “*none*–*belt.”*We then directly compared resting-state functional connectivity derived from data corrected with either RETROICOR*_belt_*or RETROICOR*_spine_*. Our ROI-to-ROI functional connectivity analysis did not reveal any significant differences.

#### 3.2.2 Functional activity

For the task dataset, we examined stimulus-related activity for data corrected with signal from each type of device. Our GLM analysis revealed a highly similar activity pattern in response to the stimulus for models containing RETROICOR*_belt_*or RETROICOR*_spine_*regressors. As illustrated inFigure 3B, we observed a widespread stimulus-related activity increase in areas overlapping the fronto-parietal network and the salience network, and an activity decrease in regions that are similar in location to the nodes of the default mode network.

Next, we investigated differences in stimulus-related activity between both denoising types. We did not observe any significant differences in stimulus-related activity between*belt*and*spine*(Fig. 3B).

#### 3.2.3 Residual noise in the resting-state dataset

We first confirmed that physiological noise modeling, indeed, reduces physiological noise in the data. To compare the amount of remaining noise after physiology correction (RETROICOR*_belt_*or RETROICOR*_spine_*) with no physiology correction (*none*), the temporal standard deviation of the denoised signal which included physiology regressors was compared with the standard deviation of the denoised signal that did not include physiology regressors. As expected, residual noise was higher without physiological noise modelling. There was a statistically significant difference between*none*and*belt*as well as*none*and*spine*throughout the whole-brain volume, with higher standard deviation values for denoising without physiology regressors (both T_55_5.73, p < 0.05, FWE corrected; seeFig. 4). Similar but less widespread differences were observed when comparing adjusted R^2^values of both models (with higher adjusted R^2^for models including physiology; data not shown).

**Fig. 4.**
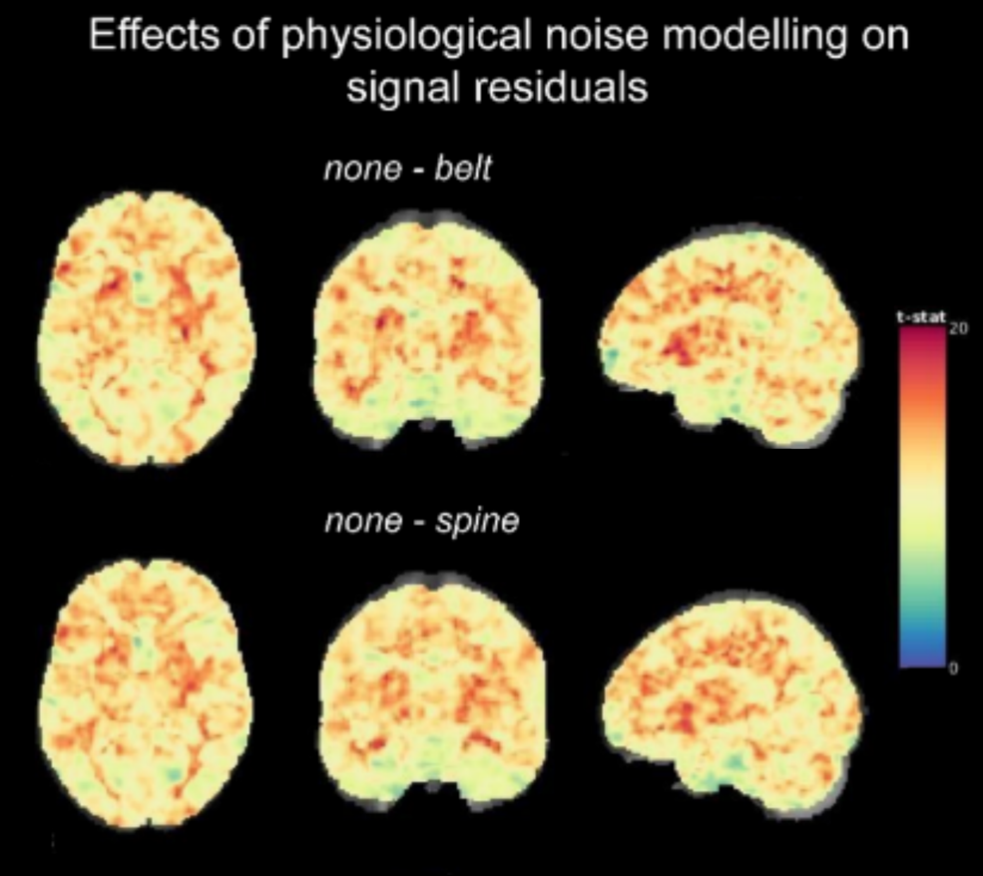
Comparison of remaining noise (standard deviation of model residuals) for*none*versus*belt*(top) and*none*versus*spine*(bottom) in the rest dataset.

To examine which device led to a more effective physiological noise removal in the resting-state data, we directly compared the standard deviation of residuals for the*belt*-denoised and*spine*-denoised data using a paired t-test. We found no significant differences between the standard deviation of the denoised signal of RETROICOR*_belt_*and RETROICOR*_spine_*at a threshold of p < 0.05, FWE-corrected. Consistent results were observed when using adjusted R^2^instead of the standard deviation of residuals (data not shown).

#### 3.2.4 Residual noise in the task dataset

Finally, we examined possible differences in the remaining noise for both denoising types in the task dataset. Again, we found no significant differences between the standard deviation of the denoised signal of RETROICOR*_belt_*and RETROICOR*_spine_*at a threshold of p < 0.05, FWE-corrected.

## 4 Discussion

Physiological noise is one of the main sources of non-neuronal noise in the BOLD signal. Recording physiological signals such as pulse and respiration during fMRI acquisition can minimise their impact on functional data by removing the main components of noise retrospectively. In this article, we compared two different respiratory signal recording devices for their overall ability to reduce physiological noise in fMRI data. To evaluate the consistency of the effects, we assessed their impact in a resting-state dataset and a task-based dataset and found no significant differences when directly comparing the two devices. In sum, our results demonstrate that the new spine coil sensor technology is as good as the conventional respiratory belt recording as a source signal for physiology denoising, but is more comfortable for participants and is less prone to artifacts.

### 4.1 No difference between belt and spine coil sensor

Our results emphasise the importance of including physiological noise correction in fMRI analyses, as there was significantly more residual noise in data without any reference-based physiological correction compared to data denoised with either RETROICOR*_belt_*or RETROICOR*_spine_*. This effect was consistent for denoising using signals from both devices and was present throughout the brain. Similarly, we found significantly higher functional connectivity in physiologically uncorrected data compared to data corrected with both*belt*and*spine*regressors. Since physiological noise is known to manifest itself as spurious correlations between voxels, remaining connectivity in the uncorrected data reflects physiological noise and indicates its efficient removal in the corrected data with both signal types. Importantly, a direct comparison of*belt*and*spine*in functional connectivity, task-based activity, and residual noise did not reveal any significant differences. Our results thus show that, given both signals have high quality and are artifact-free, the new spine coil sensor technology is well suited for physiological noise removal.

While being equally suited for physiological noise correction when the signal quality is sufficient, the spine coil sensor is additionally less prone to artifacts. In the current study, we excluded six participants who exhibited severe artifacts in the belt signal. However, less severe cases were still included in the study. This may explain the signal correspondence between both devices, which is high, but not perfect and shows some variation across participants. It is noteworthy that no participant was excluded because of the low quality of the*spine*coil sensor signal, which speaks in favor of this technology.

In the current study, we used RETROICOR, which is only one of the possible ways to model physiological noise in fMRI data (Glover et al., 2000). Although it is a highly recommended and common option, there are several other reference-based approaches that can be used for physiological noise correction. These approaches focus on different physiological parameters, for example, respiration volume per time, respiratory variation, respiratory response function, which accounts for variations in cardiac and respiratory rate, or the breathing depth and end-tidal CO_2_changes (Birn et al., 2006,^2008^;Chang & Glover, 2009). While RETROICOR may be robust to ceiling effects observed in the belt recording, respiration volume may be more sensitive to this problem, which would show up as a stronger difference between belt- and spine-derived regressors. To support and complement present results, future studies will need to ensure that the spine coil sensor performs equally well for other types of physiological noise modelling.

### 4.2 Limitations and future directions

Our study has several limitations which should be considered. First, it focused on regular breathing patterns of freely breathing individuals, showing that two devices are comparable in capturing these patterns. It is not clear whether breathing belt and spine will perform equally under special conditions, such as deep breaths, breath holds, and fast breathing. Although we tried to find episodes in our data that would be representative of such events and examine the signal consistency during those times (see SupplementaryFigs. S5 and S6), a dedicated experiment with controlled breathing would be more suitable to answer this question. This could be one future research direction.

Second, we compared spine coil sensor with a conventional pneumatic belt, which is a part of the standard Siemens equipment. It is noteworthy that the shortcomings of the pneumatic belt recordings are not generalisable to all types of respiration belts. Some belt models can minimise the risk of motion-induced artifacts, and flexible straps or transducer-based belts eliminate the issue of ceiling effects. It is therefore not clear how spine coil sensor compares to other types of respiration measurement technologies. The breathing belt used in this study is, on the other hand, like the spine coil sensor, part of the standard Siemens equipment of the on-site MRI scanner. Recordings with this belt are therefore easily accessible without purchasing extra equipment and are available in the format of Siemens physiology log files. Hence, even though better options exist, it is still important to compare these two methods.

Third, it remains unclear how participants’ weight, height, and breathing type (thoracic vs. abdominal breathing) affect the performance of both devices. Although height and weight of a participant is information that is routinely stored during an MRI exam, it was unavailable to us due to data protection policies and anonymisation procedures that are in place at our institution. Future studies can explicitly collect this information to test this relationship.

Fourth, previous studies showed that physiological functions such as cerebrovascular reactivity (CVR), which reflects the blood vessels’ adaptability to physiological factors, can be derived from breathing belt data (Pinto et al., 2021). This emphasises the variety of fields our results pertain to. Comparing CVR obtained from both devices could strongly complement these findings. Furthermore, other devices that are becoming more common for respiratory recording, such as the pulse oximeter (Addison et al., 2012;Dehkordi et al., 2018), should be compared with the breathing belt and/or spine sensors with respect to their suitability for respiration recording.

## 5 Conclusion

Our study shows that the spine coil sensor is a good alternative to the breathing belt to effectively reduce physiological noise in resting-state and task-based fMRI data. Being equally suitable for physiological noise removal, it offers additional advantages, such as increased participants’ comfort and fewer device-related artifacts.

## Data and Code Availability

Data and code are available on OSF at:https://osf.io/hr7v4/.

## Author Contributions

M.W.: Conceptualisation, Methodology, Software, Formal analysis, Investigation, Writing—original draft, and Visualisation. A.I.: Writing—review & editing, Supervision. N.Z.: Conceptualisation, Methodology, Resources, Writing—review & editing, Supervision, and Funding acquisition.

## Funding

This work was supported by the BioTechMed-Graz Young Research Group Grant to N.Z.

## Declaration of Competing Interest

The authors declare that they have no known competing financial interests or personal relationships that could have influenced the work reported in this paper.

## Supplementary Material

Supplementary Material
