# Supplementary material for: Respiration recording for fMRI: breathing belt versus spine coil sensor
Source: Imaging Neurosci (Camb). 2024 Jul 24;2:imag-2-00239. doi: 10.1162/imag_a_00239 (PMC12272212; doi:10.1162/imag_a_00239)

Supplementary Material

Respiration recording for fMRI: breathing belt versus spine coil sensor

Marilena Wilding[1,2], Anja Ischebeck[1,2], Natalia Zaretskaya[1,2]

[1] Institute of Psychology, University of Graz, Universitätsplatz 2, 8010 Graz, Austria

[2] BioTechMed-Graz, Mozartgasse 12, 8010 Graz, Austria

Figure S1. Raw signal for belt and spine for exemplary participants in the **rest dataset**. For better visibility, figures show the middle 50% of samples in the run. The complete individual time series are available at https://osf.io/hr7v4/

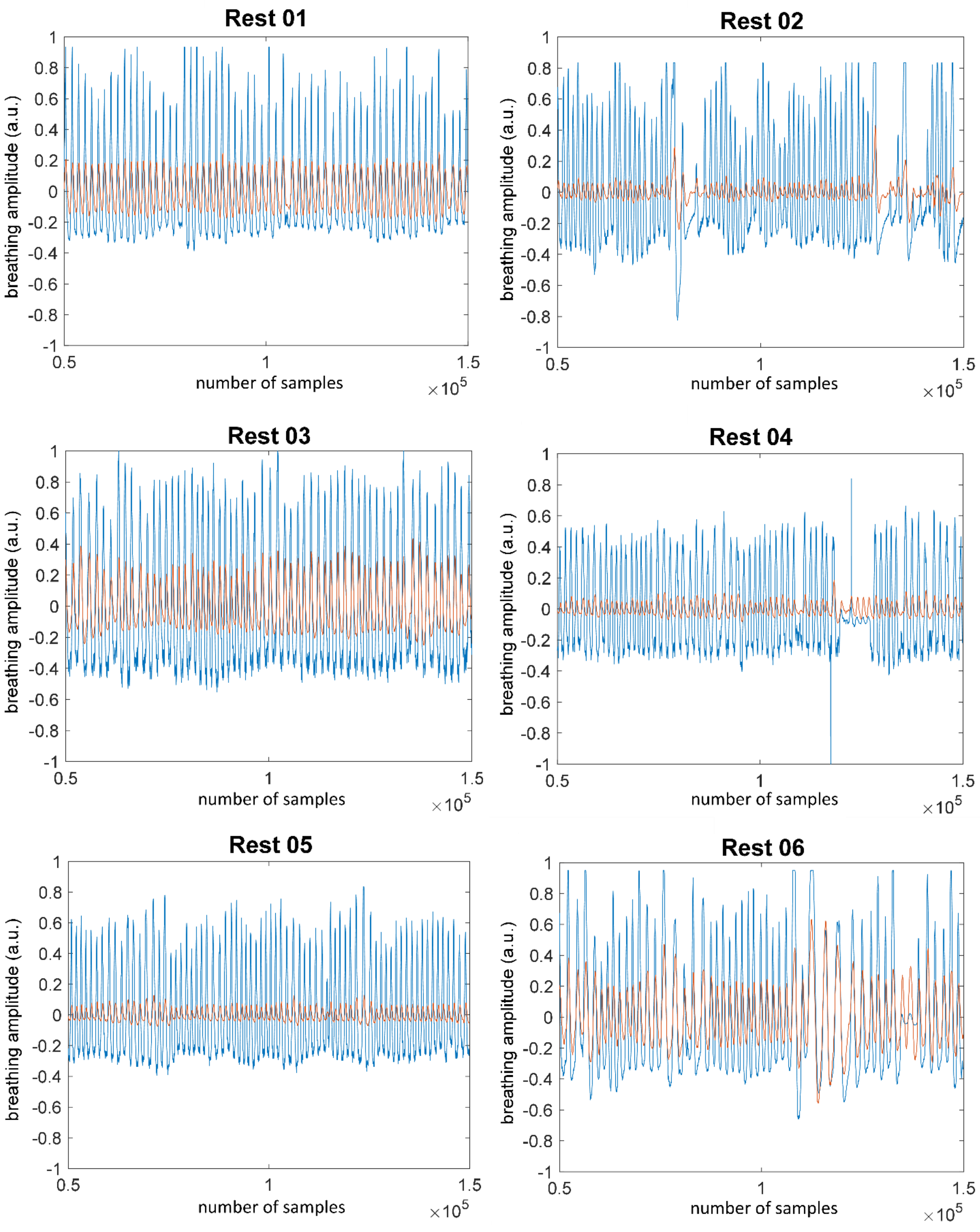

Figure S2. Raw signal for belt and spine for exemplary participants in the **task dataset**. For better visibility, figures show the middle 50% of samples in the run. The complete individual time series are available at https://osf.io/hr7v4/.

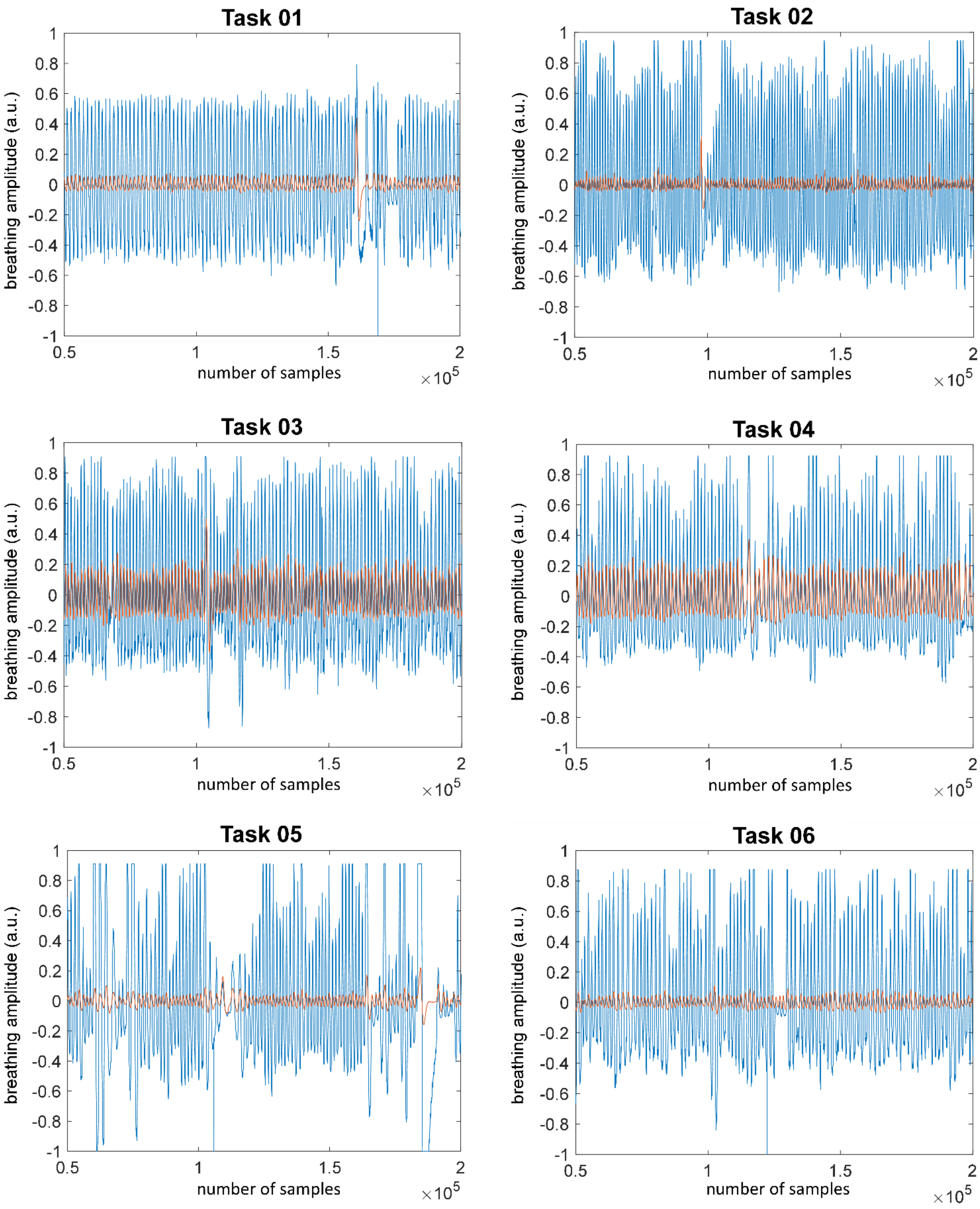

Figure S3. Power spectra of raw and z-transformed signal for belt and spine for three exemplary participants.

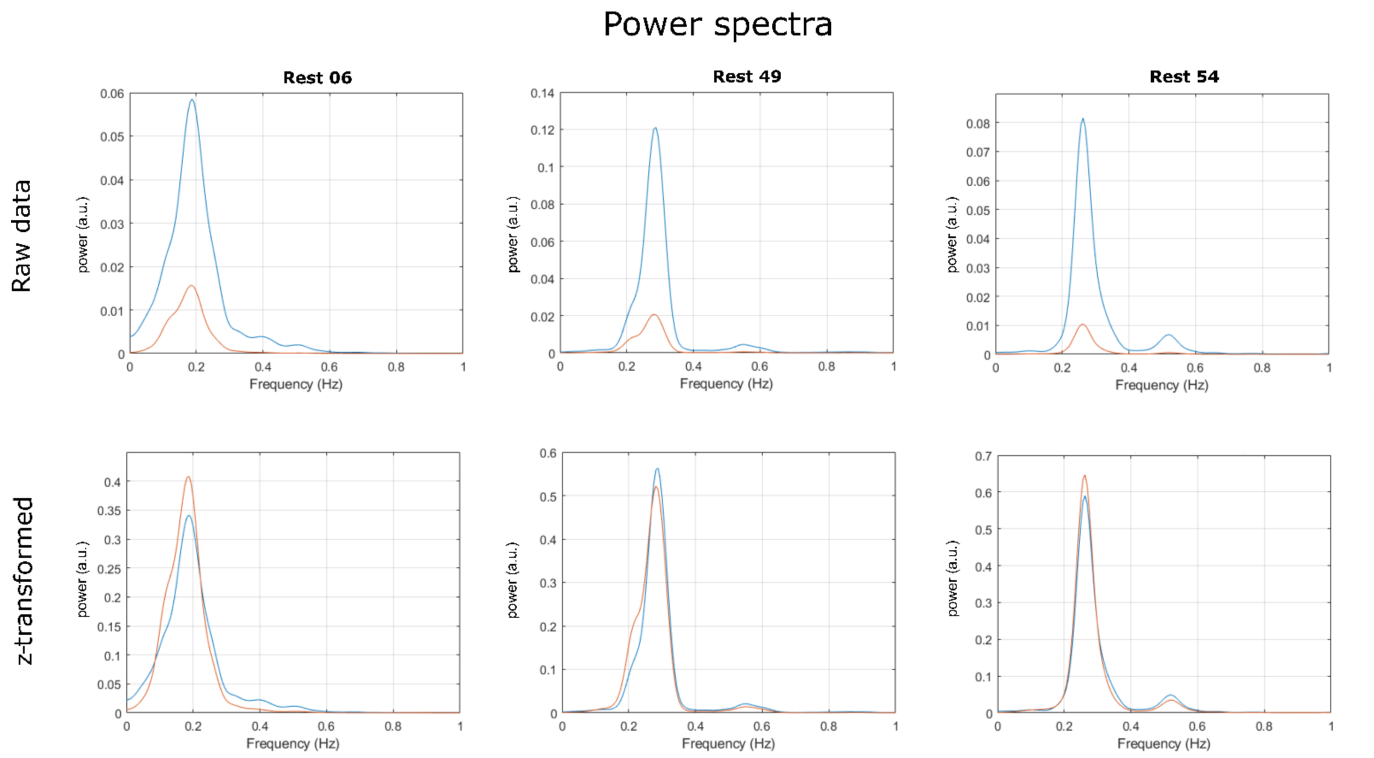

Figure S4. A. Matrices of the eight orthogonalized respiratory regressors derived from signal originating from belt or spine for 3 example participants from the rest dataset. B. Correlation between belt and spine orthogonalized regressors for each regressor column. Symbols represent individual participants. While the similarity is high for the two 1st-order terms of the Fourier expansion (regressors 1 & 2), it gradually decreases for higher-order terms.

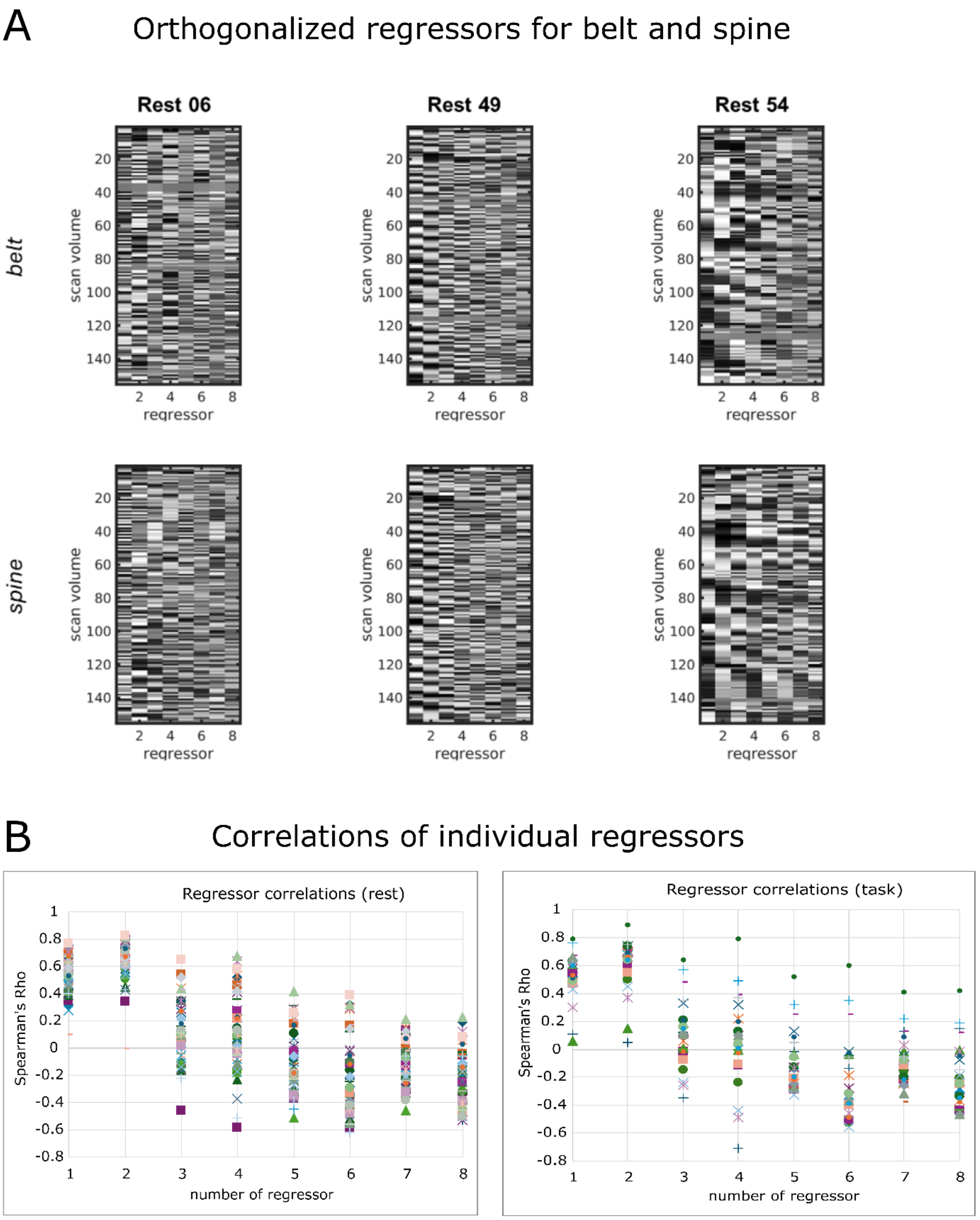

Figure S5. Deep breaths captured by belt and spine in a few exemplary subjects for each dataset. Deep breathing periods are highlighted in gray, signal clippings leading to a ceiling effect in the signal are indicated by the red arrows.

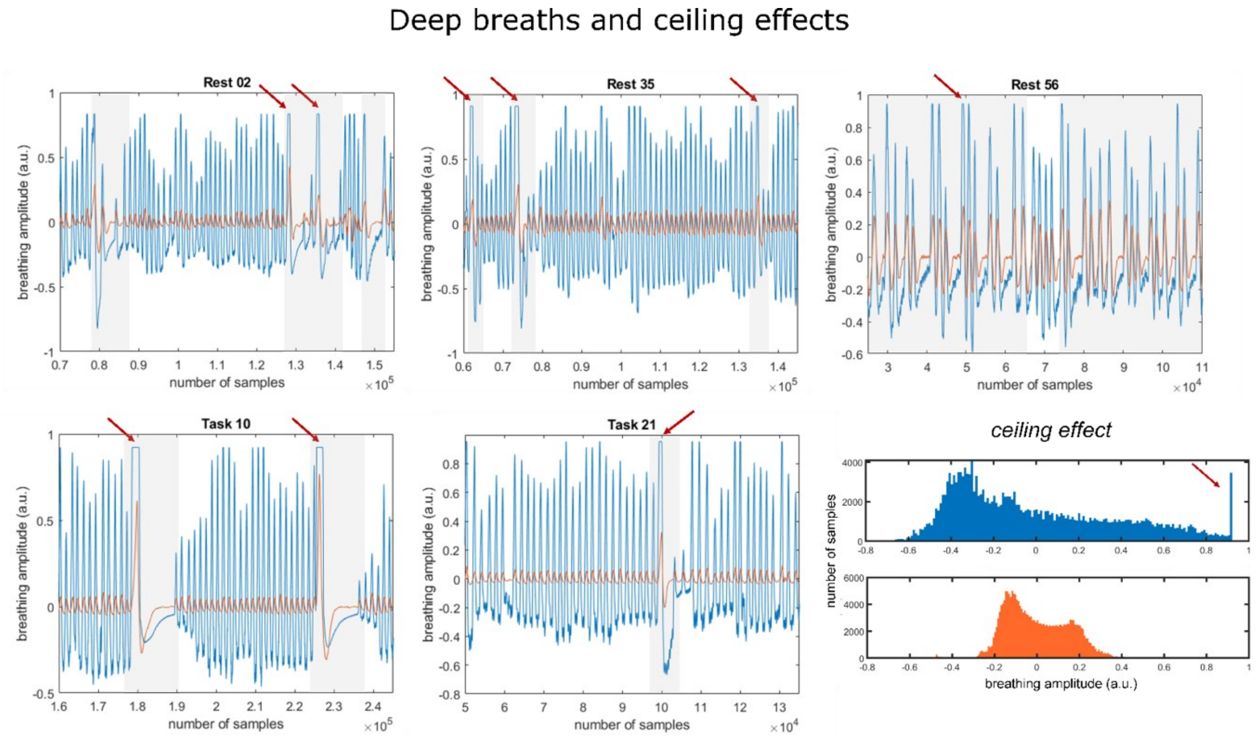

Figure S6. Short breaths captured by belt and spine in a few exemplary subjects for each dataset. Based on signal power spectra (top panel) three participants with particularly long periods of short breathing were selected (bottom panel).

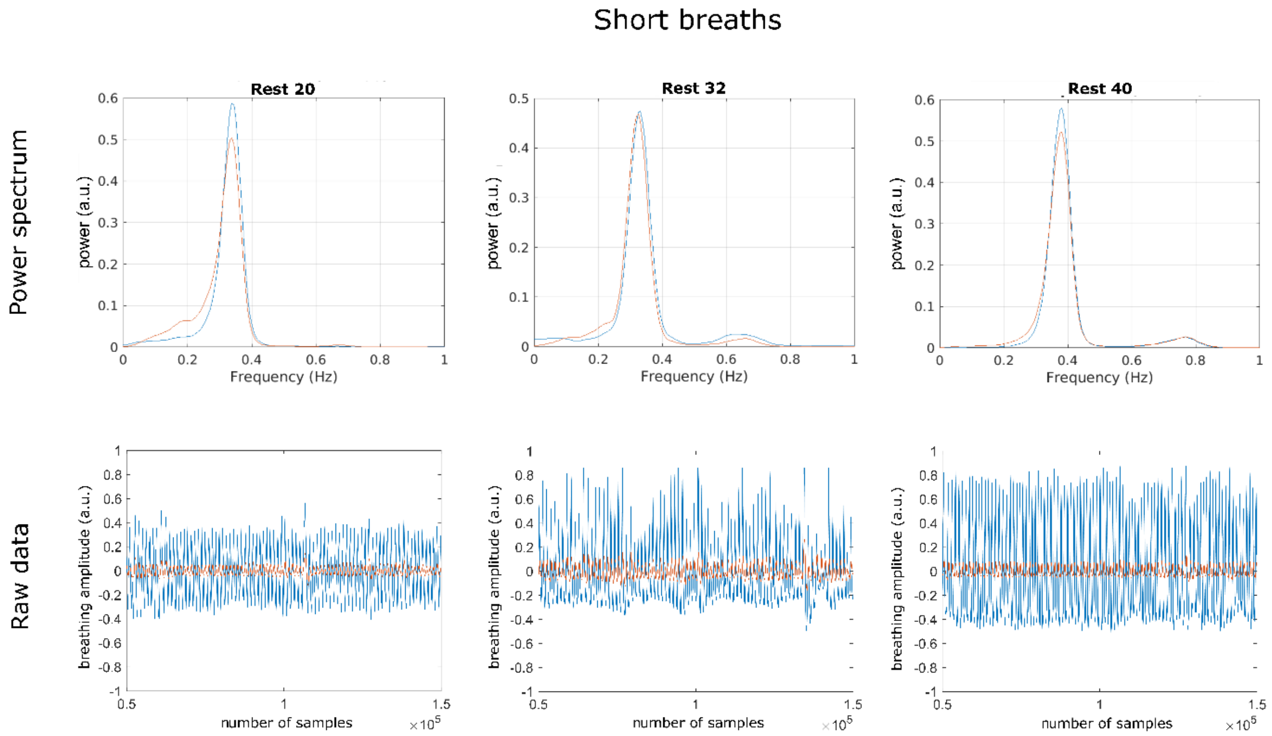

Supplement: Supplementary Material [file imag_a_00239-supp.pdf]
